# Exploiting phenotypic heterogeneity to improve production of glutathione by yeast

**DOI:** 10.1186/s12934-024-02536-5

**Published:** 2024-10-07

**Authors:** Mingzhi Xu, Cindy Vallières, Chris Finnis, Klaus Winzer, Simon V. Avery

**Affiliations:** 1 School of Life Sciences, University of Nottingham, University Park, Nottingham, NG7 2RD UK; 2 Phenotypeca, BioCity Nottingham, Nottingham, NG1 1GF UK

**Keywords:** Metabolic engineering, Phenotypic heterogeneity, Cell individuality, Yeast metabolism, Bioprocess optimization

## Abstract

**Background:**

Gene expression noise (variation in gene expression among individual cells of a genetically uniform cell population) can result in heterogenous metabolite production by industrial microorganisms, with cultures containing both low- and high-producing cells. The presence of low-producing individuals may be a factor limiting the potential for high yields. This study tested the hypothesis that low-producing variants in yeast cell populations can be continuously counter-selected, to increase net production of glutathione (GSH) as an exemplar product.

**Results:**

A counter-selection system was engineered in *Saccharomyces cerevisiae* based on the known feedback inhibition of gamma-glutamylcysteine synthetase (*GSH1*) gene expression, which is rate limiting for GSH synthesis: the *GSH1* ORF and the counter-selectable marker *GAP1* were expressed under control of the *TEF1* and GSH-regulated *GSH1* promoters, respectively. An 18% increase in the mean cellular GSH level was achieved in cultures of the engineered strain supplemented with D-histidine to counter-select cells with high *GAP1* expression (i.e. low GSH-producing cells). The phenotype was non-heritable and did not arise from a generic response to D-histidine, unlike that with certain other test-constructs prepared with alternative markers.

**Conclusions:**

The results corroborate that the system developed here improves GSH production by targeting low-producing cells. This supports the potential for exploiting end-product/promoter interactions to enrich high-producing cells in phenotypically heterogeneous populations, in order to improve metabolite production by yeast.

**Supplementary Information:**

The online version contains supplementary material available at 10.1186/s12934-024-02536-5.

## Background

Microorganisms have been extensively used to produce foods, pharmaceuticals and other beneficial substances. In response to the evolving market demands and the need for sustainability and efficiency in industrial operations, continuous improvement of product synthesis by microorganisms is desirable to achieve higher yields and/or lower production costs [1]. The development of recombinant DNA technologies, synthetic biology and metabolic engineering, alongside biosafety and high production yields, has enhanced the value of certain yeasts as cell factories for production [2]. At present, numerous valuable products, ranging from metabolites to recombinant proteins, are manufactured by yeasts, including ethanol, lactic acid, artemisinin, glutathione, insulin, glucagon and hepatitis B surface antigen [3–6]. To improve cost-effectiveness, efforts have been made to improve bioproduction in yeast over several decades.

Various biotic and abiotic factors can affect microbial production, including strain selection, medium composition and culture conditions [7–9]. One key biological problem that may impact bioproduction on an industrial scale is heterogeneity within genetically uniform cell populations [10]. This heterogeneity originates from non-genotypic cell-to-cell variation, known as phenotypic heterogeneity. Phenotypic heterogeneity is thought to be an evolutionarily selected bet-hedging strategy that cell populations benefit from *via* enhanced adaptability, resilience and survival chances in unpredictable or changing environments [11,12]. Mechanistically, phenotypic heterogeneity may arise from factors such as uneven division of cells and their constituents, heterogeneous stages of the cell cycle or cell ageing, epigenetic modifications and stochastic gene expression [11,13,14]. Studies at the single-cell level within isogenic populations of yeast and *Escherichia coli* have revealed that the noise levels of certain mRNAs and proteins between individual cells are remarkably high [15,16]. In terms of bioproduction, these variations can create a wide range of metabolite- and protein-of-interest levels among individual cells within producing populations. This phenomenon has been observed in various bioproduction processes, such as the production of free fatty acid, tyrosine and recombinant proteins [17–20], where the production levels obtained are lower than might be if all cells were in the optimal production state.

Whereas phenotypic heterogeneity can be viewed as a problem in current bioproduction processes, as described above, it may also present an opportunity to improve yield via potential enrichment of high-producing cells at the expense of low-producing cells. In studies with *E. coli*, production of free fatty acid and tyrosine was improved by using product-activated promoters to confer a growth advantage to phenotypically variant high-performing cells under antibiotic selection [20]. However, very few studies have focused on exploiting phenotypic heterogeneity for enhanced biosynthesis in yeasts. Two recent studies used biosensor-based systems to extend productive lifetimes of heterologous vanillin-*β-*glucoside and *N*-acetylglucosamine in *Saccharomyces cerevisiae* via selection of producing subpopulations [21,22]. For improved production of vanillin-*β-*glucoside, an essential gene was placed under the control of a synthetic promoter, inducible in the presence of the vanillin-*β-*glucoside precursor [21]. For improved production of *N*-acetylglucosamine, the authors engineered a conditional addiction circuit using a suicide gene *FCY1* regulated by* glmS* ribozyme that degrades its transcripts when triggered by a high level of the product intermediate [22]. There was more stable production over time in these systems compared to the non-selected controls, although the production under selection did not surpass the original control levels. This might possibly be due to the engineered control circuits responding to the target product intermediate, limiting any improvement in production beyond initial control levels. In the previous study in *E. coli*, the cell growth was regulated in response to the final products directly and there was three-fold improved production of both free fatty acid and tyrosine compared to the original level of the non-selected controls [20]. A similar type of approach using a control circuit responding to the target end-product for successfully enhancing biosynthesis in yeast remains poorly explored.

**Fig. 1:**
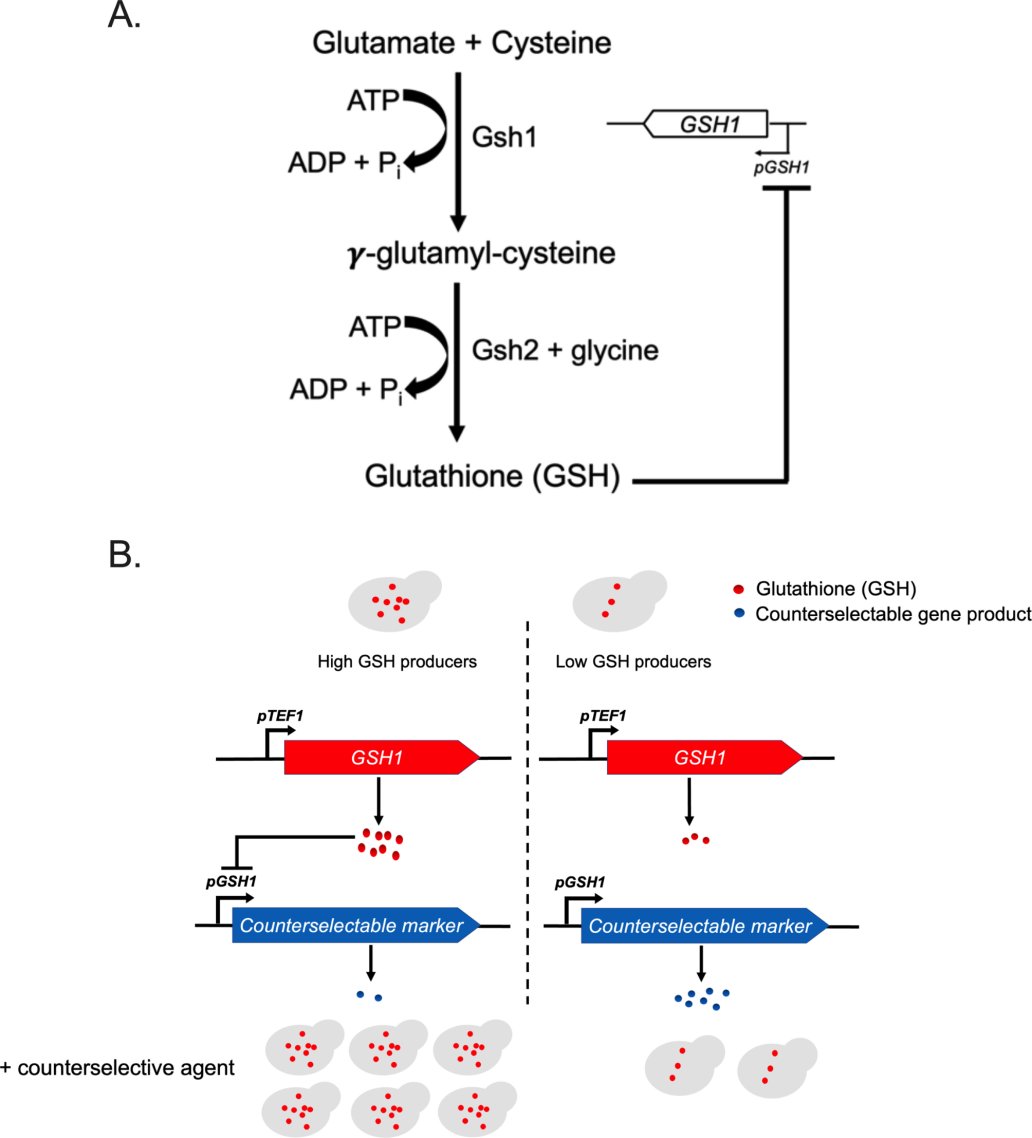
Design principle of the feedback loop for selection of high-GSH producing cells. **(A)** Glutathione biosynthesis is composed of two ATP-dependent steps: conjugation of cysteine and glutamate to form the intermediate product glutamylcysteine and combination of glutamylcysteine with glycine to form glutathione; a high level of the end-product glutathione (GSH) feedback inhibits expression from the *GSH1* promoter that otherwise drives expression of the rate-limiting enzyme for GSH synthesis. Gsh1: gamma-glutamylcysteine synthetase; Gsh2: glutathione synthetase. **(B)** The feedback loop illustrated here is designed to confer a growth advantage to high-GSH producing individual cells, enriching their proportion in the whole population in the presence of the relevant counter-selective agent

To investigate whether a higher production titre of native product can be achieved in yeast based on non-genotypic cell-to-cell variation, here we used glutathione (GSH) as an exemplar product for the following reasons: (1) the commercial production of GSH is mainly in *S. cerevisiae* due to its relatively high intracellular GSH content [23]; (2) GSH is a high-value product, with much previous effort focused on optimization of medium composition, culture conditions and genotype (e.g. via overexpression of glutathione synthetic genes) to achieve improved GSH production [8; 24–29]; (3) GSH biosynthesis in yeast is tightly controlled via a feedback repression mechanism [30; 31] (Fig. 1A), which provides a means to couple GSH level to a growth advantage. In this study, we designed a feedback inhibition loop in *S. cerevisiae* for the selection of high-GSH-producing cells based on the GSH-regulated *GSH1* promoter (*pGSH1*) and a counterselection marker. The work highlights the potential for exploiting phenotypic heterogeneity to enhance bioproduction in yeast, offering an avenue for the further development of yeast cell factories.

## Materials and methods

### Strains and culture conditions

Yeasts were maintained and grown either in YPD broth [1% yeast extract (Oxoid), 2% bacteriological peptone (Oxoid), 2% D-glucose], or in YNB broth [0.69% yeast nitrogen base without amino acids (Formedium), 2% D-glucose] supplemented as required with amino acids, uracil, 5-FOA (Melford), D-histidine (Sigma) or reduced GSH (Sigma). Where necessary, the medium was solidified with 2% (w/v) agar. Yeast cultures (10 mL) were grown in 50 mL Erlenmeyer flask from single colonies at 30 °C, 120 rev. min^− 1^ (50 mm orbit).

The starting yeast strains used in this study were the haploid *S. cerevisiae* strains BY4741 (*MAT***a**; *his3*∆*1*; *leu2*∆*0*; *met15*∆*0*; *ura3*∆*0*) and BY4742 (*MAT***a**; *his3*∆*1*; *leu2*∆*0*; *lys2*∆*0*; *ura3*∆*0*), and the isogenic diploid strain BY4743 (*MAT***a/a**; *his3*∆*1*/*his3*∆*1*; *leu2*∆*0*/*leu2*∆*0*; *lys2*∆*0*/*LYS2*; *MET15*/*met15*∆*0*; *ura3*∆*0*/*ura3*∆*0*).

*E. coli* XL1-Blue electrocompetent cells were used for cloning and plasmid amplification. The cells were grown in Luria-Bertani (LB) medium containing 100 µg/L ampicillin at 37 °C, 150 rev. min^− 1^ (50 mm orbit). Where necessary, the medium was solidified with 2% (w/v) agar. Electroporation was carried out using a MicroPulser Electroporator (BIO-RAD) at 1.8 kV. Cells were recovered with 1 mL LB medium at 37 °C for one hour before spreading to LB agar plates supplemented with 100 µg/L ampicillin.

A diploid, heterozygous yeast strain was constructed in which *URA3* was expressed as counter-selectable marker in the BY4743 background. To do that, the *URA3* open reading frame (ORF) was amplified from plasmid pCM190 (Euroscarf), *GFP* fused to *ADH1* terminator sequence (*GFP-tADH1*) and *HIS3MX6* sequence were amplified from plasmid pFA6a-GFP(S65T)-His3MX6 [32], the *TEF1* promoter (*pTEF1;* 0–408 bp upstream of the *TEF1 ORF)*, and 500-bp upstream and downstream sequences (HS) from the target homologous sequence for recombination were amplified from yeast genomic DNA. The *URA3*,* GFP-tADH1* and HS fragments for *GSH1* were ligated between the *Sac*I and *Sal*I sites of pRS315 (ATCC) via Gibson assembly using a HiFi DNA assembly cloning kit (New England Biolabs, NEB) according to the manufacturer’s instructions. Similarly, *His3MX6*,* pTEF1* and HS fragments for *pGSH1* (*GSH1* promoter) were also ligated into pRS315 using Gibson assembly. The *HS-His3MX6-pTEF1-HS* fragment was amplified and used for transformation of *S. cerevisiae* BY4741 to replace the *GSH1* promoter with the strong constitutive promoter *pTEF1* by homologous recombination, with *pgsh1*D::*HIS3MX6-pTEF1* transformants selected by growth in YNB minus histidine. The *HS-URA3-GFP-tADH1-HS* fragment was amplified and used for transformation of *S. cerevisiae* BY4742 to replace the *GSH1* ORF with *URA3-GFP-tADH1*, with *gsh1*D::*URA3-GFP-tADH1* transformants selected in YNB minus uracil. The two modified strains were crossed by co-culture in YPD broth, and a diploid heterozygous strain (i.e. *pGSH1/pgsh1*D::*HIS3MX6-pTEF1 ; GSH1/gsh1*D::*URA3-GFP-tADH1*) selected on YNB agar supplemented with leucine only.

To construct a diploid homozygous strain expressing *URA3* as the counter-selectable marker in the BY4743 background, first *pGSH1* sequence (0–585 bp upstream of the *GSH1* ORF) and HS fragments for *URA3* were amplified from yeast genomic DNA. A pRS315-*HS-pGSH1-URA3-GFP-tADH1-HS* plasmid was subsequently constructed using these and *GFP* (above) fragments, with the same Gibson approach as described earlier. *HS-His3MX6-pTEF1-HS* and *HS-pGSH1-URA3-GFP-tADH1-HS* fragments amplified from the relevant pRS315 constructs (described above) were each used to transform both strains BY4741 and BY4742, to replace *pGSH1* with *pTEF1* and inserting *URA3-GFP-tADH1* in the *URA3* locus, creating *pgsh1*D::*His3MX6-pTEF1; ura3*D::*pGSH1-URA3-GFP-tADH1* in both the BY4741 and BY4742 backgrounds. These two strains were crossed as described above to select for the diploid homozygous *pGSH1-URA3* strain (i.e. *pgsh1*D::*HIS3MX6-pTEF1*/*pgsh1*D::*HIS3MX6-pTEF1; ura3*D::*pGSH1-URA3-GFP-tADH1/ura3*D::*pGSH1-URA3-GFP-tADH1*). A homozygous control *pURA3-URA3* strain (i.e. *pgsh1*D::*HIS3MX6-pTEF1*/*pgsh1*D::*HIS3MX6-pTEF1; ura3*D::*URA3-GFP-tADH1/ura3*D::*URA3-GFP-tADH1*) was constructed in a similar way as described above.

To construct the modified homozygous strain expressing *GAP1* as the counter-selectable marker in the BY4743 background, the *KanMX* fragment was amplified from pFA6A-KanMX6 [32], and HS fragments for *pGAP1* were amplified from yeast genomic DNA and these fragments plus *pGSH1* (above) assembled into pRS315 to give pRS315-*HS-KanMX-pGSH1-HS*. Fragments *HS-His3MX6-pTEF1-HS* (described earlier) and *HS-KanMX-pGSH1-HS* were amplified and used to replace *pGSH1* with *pTEF1* and *pGAP1* with *pGSH1* in both of the BY4741 and BY4742 strains (giving genotype *pgsh1*D::*His3MX6-pTEF1; pgap1*D::*KanMX-pGSH1*), using histidine prototrophy and kanamycin resistance for selection. The two strains were crossed and the mated homozygous diploid *pGSH1-GAP1* strain (i.e. *pgsh1*D::*HIS3MX6-pTEF1*/*pgsh1*D::*HIS3MX6-pTEF1; pgap1*D::*KANMX-pGSH1/pgap1*D::*KANMX-pGSH1)* selected on YNB agar lacking histidine and supplemented with kanamycin. A homozygous control *pCYC1-GAP1* strain (i.e. *pgsh1*D::*HIS3MX6-pTEF1*/*pgsh1*D::*HIS3MX6-pTEF1; pgap1*D::*KANMX-pCYC1/pgap1*D::*KANMX-pCYC1)* was constructed in a similar way as described above with the *pCYC1* promoter (0–300 bp upstream of the *CYC1* ORF) amplified from yeast genomic DNA. Yeast transformations were by the lithium acetate/single-stranded carrier DNA/polyethylene glycol method [33] and appropriate deletion/integration was confirmed by PCR and gel electrophoresis using standard procedures [34].

## Growth measurement

Growth of yeasts was followed using a Synergy HTX Multi-Mode Reader. In brief, 5 µL of overnight culture in YNB was transferred to wells of 96-well microtiter plates containing 95 µL of YNB medium supplemented (e.g. with 5-FOA or D-histidine) as necessary for growth and selection. The OD_600_ was measured every 15 min during continuous linear shaking (1096 cycles min^− 1^, 1 mm orbit) at 30 °C for 24 h. The specific growth rate was calculated from the 3-h time window over which exponential growth rate was maximal according to the appropriate OD_600_ determinations.

## Quantification of cellular glutathione

Relative GSH levels in cells were determined by colorimetric assay of the reduction of 5,5-dithio-bis-(2-nitrobenzoic acid) (DTNB) (Sigma), according to the protocol from Rahman et al. [35]. Samples of cells were prepared by diluting exponential phase cells (OD_600_ 1.5–2.0) to an OD_600_ of 1.0 before washing twice using PBS. The cells were harvested by centrifugation (1000 x *g*, 2 min) and resuspended in lysis buffer (5% w/v 5-sulfosalicylic acid in 0.1 M potassium phosphate buffer with 5 mM EDTA (KPE buffer [35])) together with 425–600 μm-diameter acid-washed glass beads (Sigma) at a buffer/bead ratio of 1:2 by volume. Lysis of cells was performed with a homogenizer (Precellys Evolution) for six cycles of 20 s each at 6500 rev. min^− 1^, with 1 min pauses between cycles. The samples were centrifuged (10,000 *g*, 10 min) and 20 µL of the supernatants plus 60 µL of 0.66 mg/mL DTNB solution, 60 µL of 0.66 mg/mL β-NADPH (Sigma) and 60 µL of 3.33 units/mL glutathione reductase (Sigma) were used for DTNB assay. The colour change was measured at 412 nm using a Synergy HTX Multi-Mode Reader. The amount of glutathione from cells was read from a standard curve via the rate of change in the absorbance (∆A_412nm_ min^− 1^).

## Flow cytometry

Exponential phase cells (OD_600_ ~ 1.5–2.0) in YNB were diluted to OD_600_ 0.5, then 1 mL samples washed twice in PBS before resuspension in 1 mL PBS. Cells were examined for cell size (forward scatter) and GFP fluorescence distributions using a Becton Dickinson FACSCanto™ Flow Cytometer (BD Biosciences) fitted with a blue filter; excitation was at 488 nm with emission measured at 530 nm.

## RNA extraction, cDNA synthesis and real-time quantitative PCR (RT-qPCR)

Total RNA was isolated from cells using a TRI Reagent kit (Sigma) according to the manufacturer’s instructions. RNA concentrations in preparations were measured by spectrophotometry (DeNovix). Isolated RNA was purified using DNase I (Sigma) before conversion to cDNA using the GoScript Reverse Transcription system (Promega). cDNA was then used as template for RT-qPCR with a QuantStudio 3 Real-Time PCR system (Applied Biosystems) using PowerUP SYBR Green Master Mix (Applied Biosystems) and 500 nM of target gene specific forward and reverse primers, using the recommended fast cycling mode. Standard housekeeping genes in *S. cerevisiae*, *ACT1* and *UBC6*, were used as reference genes. Cycle threshold (Ct) values of target genes were calibrated to the Ct for the corresponding genes obtained for samples from the control *pCYC1-GAP1* strain and normalised to the geometric mean of the reference genes for relative quantification using the comparative Ct method [36].

### **Statistical analyses**

Data were typically expressed as the mean of at least three biological replicates ± standard error of the mean (SEM). Significant differences were determined by analysis with one-way or two-way ANOVA, using GraphPad Prism 9 software.

## Results

### Construct design for selecting high-GSH cells

As cellular GSH is known to feedback inhibit expression from the *GSH1* promoter (*pGSH1*) [30], we designed a feedback loop for enrichment of high-GSH producing cells within clonal yeast cultures (Fig. 1B). The construct design supports expression of the *GSH1* ORF under control of the *TEF1* promoter, while a counter-selectable marker is expressed under control of the GSH-regulated *pGSH1*. The rationale was that a high cellular GSH level should downregulate expression (from *pGSH1*) of the counter-selectable gene product, therefore providing a means to counter-select low GSH cells (i.e. those expected to express higher levels of counterselectable marker) by introduction of the relevant counter-selective agent to cultures. The net effect should be a relative enrichment of high-GSH producing cells in the population. *pTEF1* was chosen for bypassing GSH-regulated *GSH1* expression as *pTEF1* is a relatively strong promoter, commonly used in yeast expression systems.

### *URA3* is not a suitable counter-selectable marker in this system

We initially used *URA3* as the counter-selectable marker in the construct, for counterselection based on sensitivity of *URA3-*expressing cells to the drug 5-fluoroorotic acid (5-FOA) [37]. The constructed *pURA3-GSH1* strain was heterozygous at the *GSH1* locus, with one allele expressing the *URA3* ORF under *pGSH1* control and the other expressing *GSH1* under *pTEF1* control (FigureS1**A**). Significant increases in cellular GSH level were detected in cultures grown at 100 µg/ml or 150 µg/ml 5-FOA, but not at 50 µg/ml 5-FOA (Figure S2A). Examination of cultures including by flow cytometry showed that the cells (Figure S2B) tended to form clumps in the presence of 150 µg/ml 5-FOA, which could skew the OD-based normalization of cellular GSH level measurements. Moreover, culture growth was slowed at 150 µg/ml 5-FOA (Figure S2C). Therefore, 100 µg/ml 5-FOA was the concentration selected for further experiments.

To investigate whether the increased GSH level at 100 µg/ml 5-FOA (Figure S2A) was stable during longer-term cultivation of the modified strain, cellular GSH content was monitored every ∼10 generations during sequential batch cultivation in flasks at 100 µg/ml 5-FOA (after every ~ 10 generations, aliquots of overnight culture were transferred to fresh medium giving OD ∼0.01 and re-incubated). The GSH level increased over the first ∼20 generations (two transfers), but subsequently declined and after ∼60 generations (six transfers) had returned close to that of the control, wild-type strain cultured in the same conditions (Figure S3A). The genotype of the modified strain at the *GSH1* locus was checked by PCR after 50 generations’ growth in the presence of 100 µg/ml 5-FOA. In eight of ten colonies tested, the introduced *URA3* gene could no longer be detected (Figure S3B), and none of the negative clones grew on minimal agar without added uracil. This suggested a potential reversion. Such apparent loss of heterozygosity can arise by mitotic crossover during cell replication [38], for example, and would confer a strong advantage in the presence of 5-FOA in cells where *URA3* was lost. To address this problem an *S. cerevisiae* strain with *pTEF1-GSH1* at the *GSH1* locus on both alleles, and *pGSH1-URA3* at the *URA3* locus on both alleles was constructed (strain designated ‘homozygous *pGSH1-URA3’* in the following context) (Figure S1**B**). With the homozygous *pGSH1-URA3* strain, a ~ 50% increase of GSH cellular content was observed in cultures supplemented with 100 µg/ml 5-FOA, and this increase was relatively stable compared to that of the previous heterozygous strain (Figure S4A). To test whether this increase was due to selection of phenotypically high-GSH producers rather than a generic response (e.g. stress response) to 5-FOA, a control strain was constructed, designated *pURA3-URA3* in the following context, which retained *pTEF1-GSH1* but in which *URA3* was under the control of its native promoter *pURA3* (**Figure ****S1****C**). Despite the absence of *pGSH1*-driven *URA3* expression in the *pURA3-URA3* strain, cultivation in the presence of 5-FOA again gave a significant, ~ 50% increase in GSH level (Figure S4B). In addition, measurement of expression of *URA3* in the *pGSH1-URA3* strain according to levels of GFP-tagged Ura3 revealed a significant upregulation by 5-FOA (Figure S4C). This was unexpected as a high GSH level from selection of phenotypically high GSH producers in the *pGSH1-URA3* strain population should inhibit the expression of Ura3-GFP when under control of the GSH-regulated *pGSH1*. Because of the above evidence for selection of potential revertants and of *pGSH1* upregulation upon 5-FOA treatment (implying a generic response), it was concluded that *URA3* is not a suitable counter-selectable marker in this system for selection of phenotypically high-GSH producers.

### *GAP1* as an alternative counter-selectable marker in this system

**Fig. 2:**
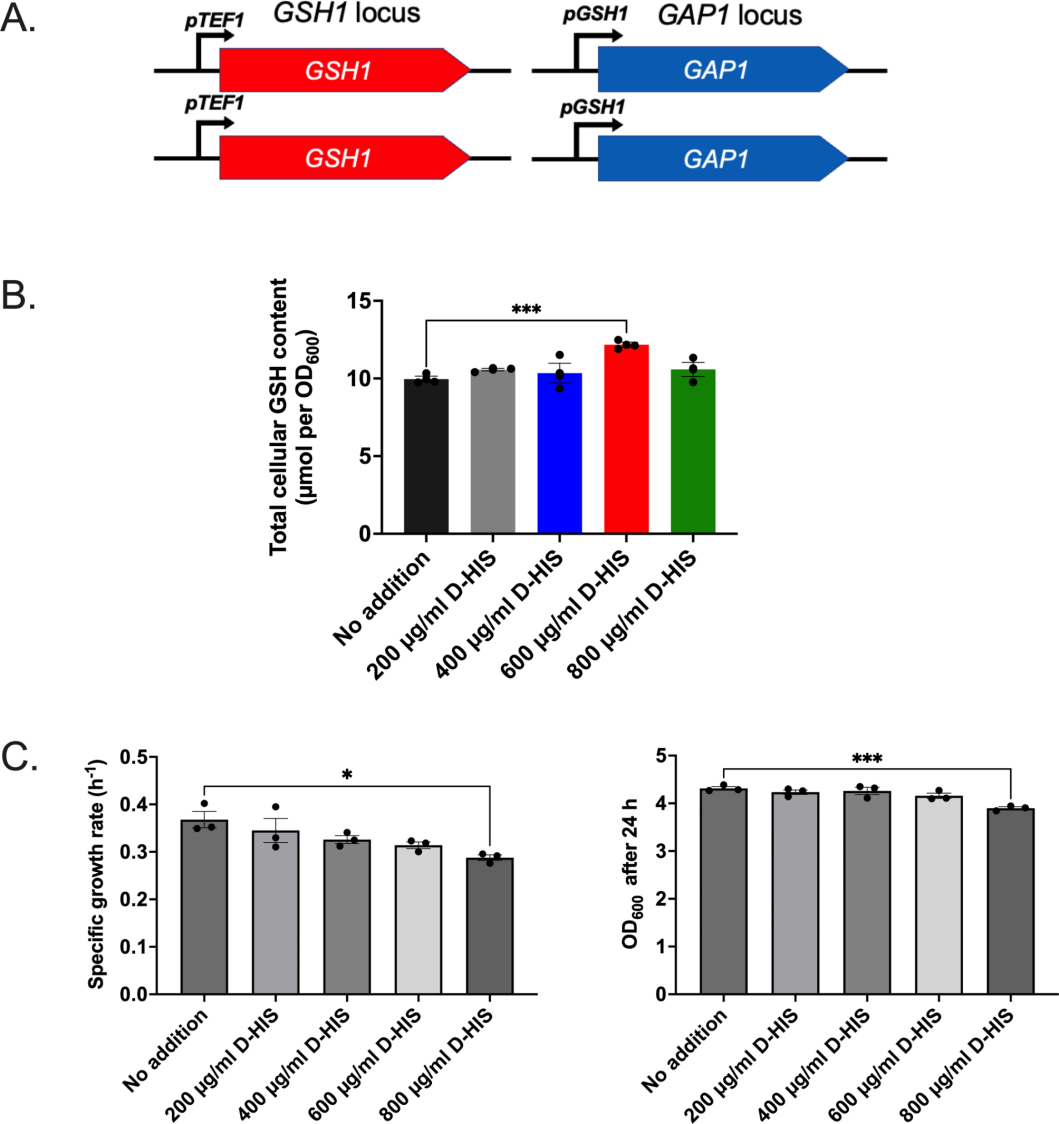
Use of *GAP1* in the counterselection system. **(A)** Homozygous construct design with *GAP1* expressed under *pGSH1* control at the *GAP1* locus, the modified strain designated ‘*pGSH1-GAP1*’. **(B)** Total cellular GSH contents of the *pGSH1-GAP1* strain after cultivation for 24 h in YNB medium supplemented with D-HIS at the indicated concentrations. **(C)** Growth rate and OD_600_ after 24 h for the *pGSH1-GAP1* strain in YNB medium supplemented with D-HIS at the indicated concentrations. Data represent mean values ± SEM of three biological replicates. *, *p* < 0.05; ***, *p* < 0.001

*GAP1* is a native yeast gene encoding a general amino acid permease [39]. Cells expressing Gap1 can be counter-selected using D-histidine (D-HIS), which is toxic and imported exclusively by Gap1 [40]. Therefore, as an alternative to *URA3* (above), a strain (designated *pGSH1-GAP1* in the following context) was constructed for using *GAP1* as the counter-selectable marker to enrich phenotypically high-GSH producers, via replacement of the native *pGAP1* promoter with *pGSH1* (Fig. 2A). *GAP1* expression is naturally repressed by rich nitrogen sources such as ammonium sulphate and glutamine and is induced in the presence of a poor nitrogen source [41]. A poor nitrogen source can result in a low growth rate, which itself might affect the rate of GSH synthesis non-specifically. However, in the present construct, *GAP1* expression being under the control of *pGSH1* is not subject to the repressive effect of nitrogen on *pGAP1*, enabling the use of ammonium sulphate in the cultures.

The GSH level was examined in the *pGSH1-GAP1* strain after culture (with ammonium sulphate) in the presence of a range of D-HIS concentrations (Fig. 2B). The total cellular GSH level was significantly increased at 600 µg/ml D-HIS. A further increase in D-HIS to 800 µg/ml returned the GSH close to the control level and it was noted that specific growth rate of cultures and OD_600_ after 24 h were significantly decreased at this D-HIS concentration (Fig. 2C).

**Fig. 3:**
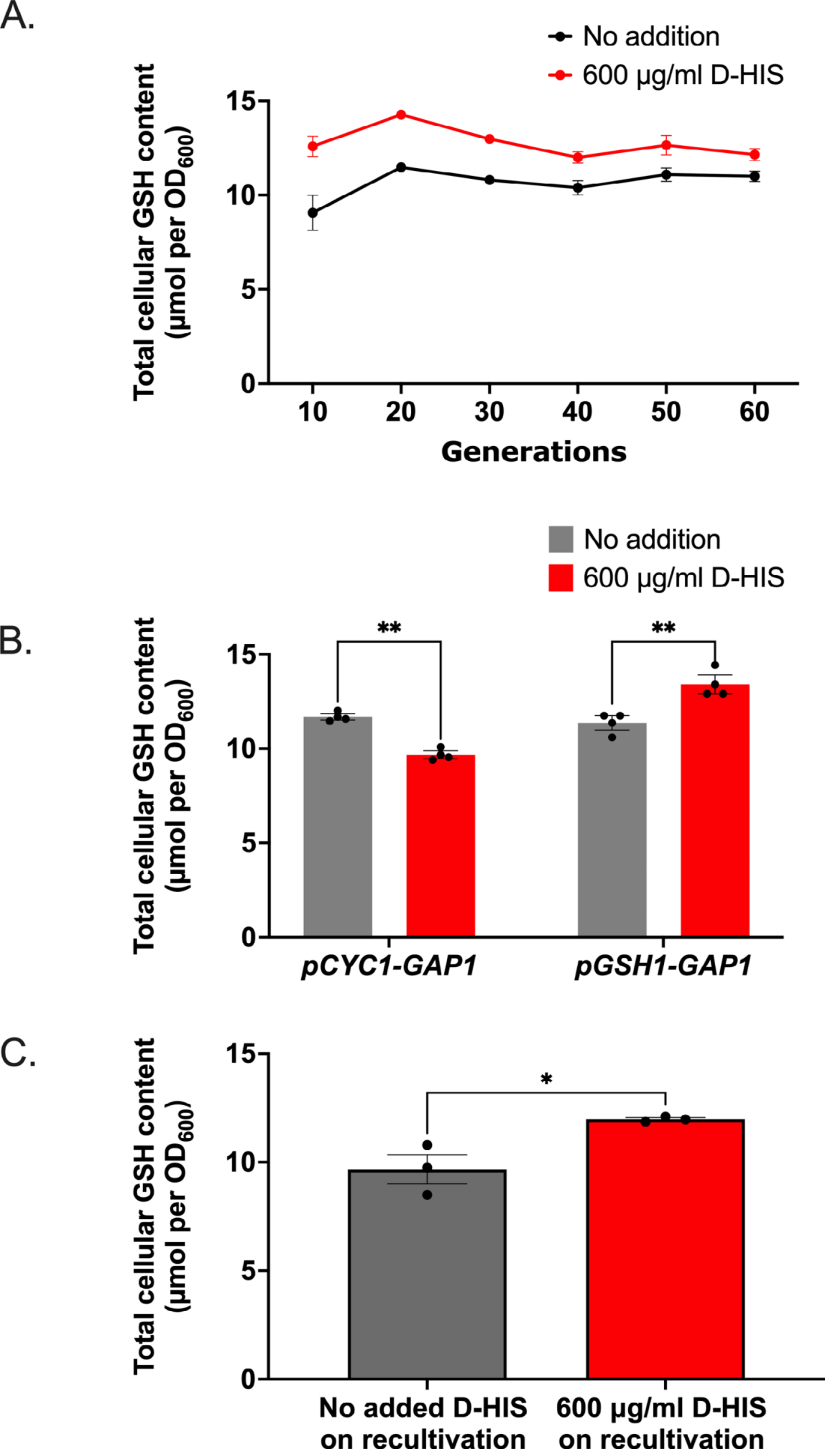
Long-term cellular GSH level under D-HIS selection in *pGSH1-GAP1* expressing *S. cerevisiae*. **(A)** Long-term cellular GSH levels in the *pGSH1-GAP1* strain (as shown in Fig. 2A), during sequential daily subculture to fresh YNB medium ± 600 µg/ml D-HIS. **(B)** The high GSH content of the *pGSH1-GAP1* strain cultured with D-HIS is absent in a control strain, *pCYC1-GAP1*. **(C)** Lack of heritability of the high GSH-producing phenotype; the *pGSH1-GAP1* strain after 60 generations’ cultivation with D-HIS was cultured without D-HIS for 24 h, then recultivated with or without D-HIS for 24 h. Data represent mean values ± SEM of three biological replicates. *, *p* < 0.05; **, *p* < 0.01

To investigate whether the increase in GSH level at 600 µg/ml D-HIS was stable during long-term cultivation, GSH content was monitored during sequential batch cultivation up to ∼60 generations. Unlike the similar earlier experiment with (heterozygous) *pGSH1-URA3* modified cells (Fig. S3A), an elevated cellular GSH level was sustained in the D-HIS supplemented *pGSH1-GAP1* strain, although the size of the effect did decrease a little over time (Fig. 3A). To exclude the possibility that the increase in GSH level could reflect a generic response to D-HIS, a control strain (designated *pCYC1-GAP1* in the following context) was constructed with *GAP1* under the control of the constitutive promoter *pCYC1.* (*GAP1* expression from its native promoter would not serve as a good control here because *pGAP1* is repressed by the ammonium sulphate N-source and so D-HIS would not be taken up by the cells). The *CYC1* promoter was selected for this as in YEPD culture it supports similar, only slightly stronger expression than *pGSH1* (but much stronger expression than *pGAP1*), according to transcriptomic data from YEPD culture [42]. Therefore, both the control strain (*pCYC1-GAP1*) and the modified strain (*pGSH1-GAP1*) are expected to show comparable levels of Gap1 expression and D-HIS uptake. In marked contrast to the *pGSH1-GAP1* strain, the control strain showed a decrease in GSH level when cultured with D-HIS (Fig. 3B). This opposed the possibility that a generic response to D-HIS could explain the increased GSH level seen in the *pGSH1-GAP1* strain with D-HIS. In addition, to allay the concern that results could reflect emergence of mutants in the population, we assayed heritability of the high-GSH phenotype following culture with D-HIS. The D-HIS treated cells at 60 generations were cultivated without D-HIS for 24 h and then recultivated with or without D-HIS. The D-HIS treated cells reproduced the high GSH phenotype only when re-cultured with D-HIS (Fig. 3C). In conjunction with the fact that the high-GSH phenotype was evident within as little as 10 generations of culture with D-HIS (Fig. 3A), the evidence did not support mutation and selection as an alternative mechanism to explain the phenotype.

**Fig. 4:**
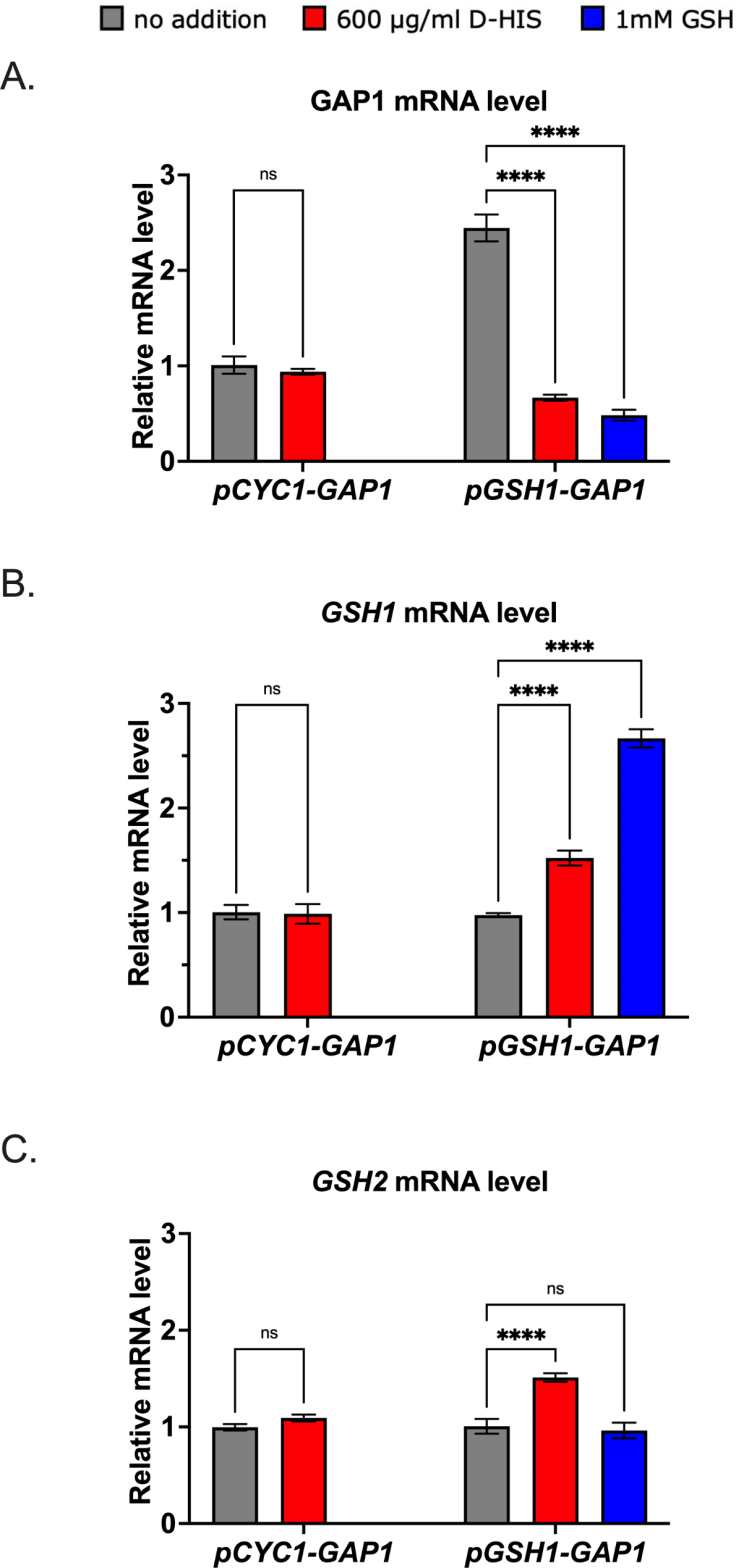
Effect of D-HIS on *GAP1*,* GSH1*, and *GSH2* mRNA levels in *pGSH1-GAP1* expressing *S*. *cerevisiae.* The *pGSH1-GAP1* strain was cultured for 24 h in YNB medium with or without 600 µg/ml D-HIS. In parallel assays, a *pCYC1-GAP1* strain was used as a negative control and the *pGSH1-GAP1* strain cultivated with 1mM GSH a positive control. The mRNA levels of *GAP1 ***(A)**, *GSH1 ***(B)**,* GSH2 ***(C)** in RNA extracts were determined by RT-qPCR. Values shown are means ± SEM of three biological replicates. ****, *p* < 0.0001; ns, not significant

To further support the observations and conclusions, expression levels of the counter-selectable gene *GAP1* and genes involved in GSH synthesis were determined by RT-qPCR in the *pGSH1-GAP1* strain. In keeping with the selection of high GSH producers which should have a reduced *pGSH1* expression level, the *GAP1* mRNA level was markedly lower during cultivation with D-HIS selection (Fig. 4A). Furthermore, expression of both the glutathione synthesis genes *GSH1* and *GSH2* was greater in the D-HIS-selected population, consistent with these cells’ higher production of GSH (Fig. 4B **and C**). The *pGSH1-GAP1* strain treated with 1 mM GSH as a positive control also showed lower *GAP1* transcript levels than the no-addition control (Fig. 4A). There was also an increase in *GSH1* transcript level in the GSH-supplement condition, suggesting the possibility of *pTEF1* regulation by relatively high (≥ 1 mM) extracellular GSH. In the *pCYC1-GAP1* negative-control strain (i.e. in the absence of *pGSH1-*driven *GAP1* expression), cultivation in the presence of D-HIS had no influence on the expression of *GAP1*,* GSH1* or *GSH2* (Fig. 4). The evidence collectively indicated that the high GSH level achieved in the *pGSH1-GAP1* strain cultivated with D-HIS resulted from the selection of phenotypically high-GSH producers.

## Discussion

Cell-to-cell phenotypic variation is commonly observed in any genetically uniform cell population. In biomanufacturing processes, high-producing cell subpopulations may have fitness disadvantages compared to low-producing cells due to the higher metabolic burden, so they may be less active, leading to lower overall product formation. A selection pressure for low-producing mutants could also be important over time. The present study has described a potential approach to push phenotype selection in yeast in the opposite direction by imposing a penalty on low producers. Therefore, the work capitalises on the existing phenotypic heterogeneity for enhancing biosynthesis, rather than stabilizing biosynthesis for longer in yeast [21,22], where previous related work that’s similar to ours has been limited to bacteria [20]. GSH was used as an exemplar product, as the heterogeneity in GSH content within yeast populations has been demonstrated and the regulation of GSH in yeast offers a framework for the selection system [30,43]. The heterogeneity in GSH content within a yeast cell population is driven at least partly by metabolic oscillations and transcriptional regulatory mechanisms [43].

The use of a counterselection system to select phenotypically high-producing cells, as in the present study, may critically depend on an appropriate choice of counter-selectable marker gene. This was exemplified here by the fact that increased GSH production in the presence of 5-FOA (counter-selective for *URA3* expression) appeared to be a non-specific response to 5-FOA, evident also in the control strain expressing *URA3* behind *pURA3*, rather than selection based on phenotypic heterogeneity as intended in this proof-of-principle study. On the other hand, *GAP1* did prove a suitable counter-selectable marker for selection of cell subpopulations producing high GSH. The ~ 18% increase in cellular GSH content of cultures subject to this selection system provides an encouraging starting point for further optimizing production in yeast. In the case of the GSH product, for example, the counter-selection system could complement or further enhance the increased GSH content that is attainable through gene knockout or overexpression approaches [43,44]. Moreover, the concentration of GSH in yeast is significantly influenced by the availability of nutrients in the growth medium [27–29]. Optimization of the medium might also further increase the GSH content attainable in the system.

When expressing *URA3* heterozygously at the *GSH1* locus, a loss of heterozygosity became evident over time under 5-FOA selection, likely attributable to mitotic crossovers. Mitotic recombination events in yeast are considered to be associated with DNA repair and genome evolution [45]. The present results support a role as a survival strategy, as rapid loss of heterozygosity and of the *URA3* marker rendered cells insensitive to 5-FOA within a few generations. Consequently, the emerging homozygous *ura3* mutants could dominate the population within 50 generations of culture with 5-FOA. *URA3/GSH1* heterozygosity was evidently an unstable state for yeast in this condition.

Previous studies showed that production lifetimes of products vanillin-*β-*glucoside and N-acetylglucosamine in yeast can be extended by controlling the population heterogeneity via control circuits responding to the target product intermediates, so the product yield was increased when measured in the long term [21, 22]. In the present study, we demonstrated that biosynthesis of native products can be enhanced, in this case, by counter-selection of low-producing cell subpopulations in a system responding directly to the target product. Additionally, this enhancement could also be seen in the short term (i.e. within 10 generations). In experiments with *E. coli*, a similar type of approach as ours gave approximately three-fold enhancement of production of free fatty acid and tyrosine [20], versus an ~ 18% increase in GSH production in the present study with yeast. Differences in the degree of cell-to-cell variation in product levels (i.e. on which selection can be exerted through these approaches) could be one factor that accounts for such differences. Furthermore, the *E. coli* strains into which the product-responsive circuits selecting nongenetic high producers were introduced had previously been engineered for overproduction of fatty acid and tyrosine, through gene knockouts, overexpression and/or heterologous gene expression [20]. In contrast, the constructs in the present study were introduced to a wild-type yeast strain. As suggested above, future studies could introduce the counterselection system to yeast strains engineered for GSH overproduction to investigate the potential for further enhancement in those backgrounds. Moreover, the counter-selection agent used in this system may also affect the level of increased GSH that is attainable. In the control strain expressing *GAP1* under the control of a constitutive *CYC1* promoter, the cellular GSH level was decreased to some extent when cultured with D-HIS (Fig. 3B). This suggests that D-HIS exposure may non-specifically decrease the cellular GSH concentration, so resulting in an underestimation of the increase in cellular GSH level potentially attainable with other counter-selection systems. Finally, the GSH product exerts regulatory effects not only at the transcriptional level but also at the enzymatic level [46]. In other words, a high concentration of glutathione not only inhibits expression from *pGSH1* but also can partly inhibit the activity of Gsh1 enzyme. Therefore, a high level of cellular GSH may still partly feedback-inhibit even if the *GSH1* is under the control of a constitutive promoter *pTEF1*, i.e., by inhibiting Gsh1 enzyme activity.

One limitation of the feedback control system used here for potential application in biomanufacturing is its use for a natural product regulated by feedback inhibition. To extend its use, a co-expression system that allows coordinated expression of two genes from the same promoter provides a potential solution. For example, another gene of interest (GOI) could be linked to *GSH1* under the control of a constitutive promoter (e.g. *pTEF1*) and the high GOI expressing cells may be simultaneously selected with the high GSH producers. Such co-expression to produce separate proteins can be achieved, for example, using an internal ribosome entry site (IRES) or a self-cleaving peptide to link the genes [47]. A previous study demonstrated that bicistronic expression of RFP and GFP was at a comparable level as their monocistronic expression using a 2 A peptide sequence from the equine rhinitis B virus to link the two gene sequences [48]. Adaptation of approaches like this could allow heterologous protein production to be linked to the production of glutathione or alternative native product by individual cells and, therefore, subject to similar counterselection against low-producing cells as described here. Currently, the counter-selectable markers for yeast are limited and the counter-selecting agents can be relatively expensive [49], which may be a limit for industrial-scale applications. Costs may come down or future development of new markers or selective agents may help to address this concern, but it should also be borne in mind that while the GSH feedback system was convenient for proof of principle in this study, the door is open to explore other regulatory systems of yeast which may prove more efficacious for enrichment of high-producing individuals including, potentially, for commercial exploitation.

## Electronic supplementary material

Below is the link to the electronic supplementary material.

Supplementary Material 1

## Data Availability

All data generated or analysed in this study are included in the article, supplementary files, or available on request.
