# Supplementary material for: Exploiting phenotypic heterogeneity to improve production of glutathione by yeast
Source: Microb Cell Fact. 2024 Oct 7;23:267. doi: 10.1186/s12934-024-02536-5 (PMC11457410; doi:10.1186/s12934-024-02536-5)
Supplement: Supplementary file 1 — Supplementary Material 1 [file 12934_2024_2536_MOESM1_ESM.docx]

**SUPPLEMENTARY MATERIAL**

**Exploiting phenotypic heterogeneity to improve production of glutathione by yeast**

Mingzhi Xu, Cindy Vallières, Chris Finnis, Klaus Winzer, Simon V. Avery

Supplementary Material Content:

Figure S1

Figure S2

Figure S3

Figure S4


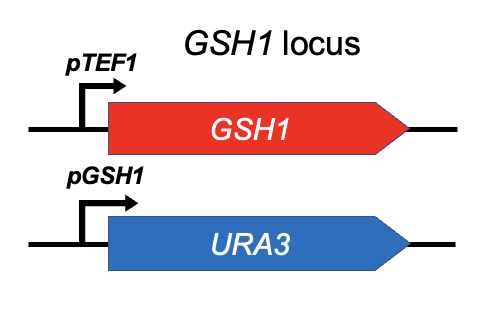

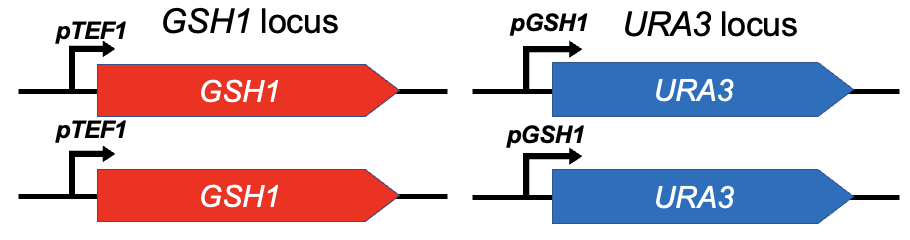


**A.**

**B.**

**Figure S1: Use of *URA3* in the counterselection system.** (A) Construct design in the ‘heterozygous *pGSH1-URA3’* strain with *URA3* expressed as one allele at the *GSH1* locus and *GSH1* under *pTEF1* control at the other allele. (B) Construct design in the ‘homozygous *pGSH1-URA3’* strain with *GSH1* under *pTEF1* control at the *GSH1* locus and *URA3* under *pGSH1* control at the *URA3* locus. (C) Construct design in the ‘control *pURA3-URA3’* strain with *GSH1* under *pTEF1* control at the *GSH1* locus and *URA3* under *pURA3* control at the *URA3* locus.


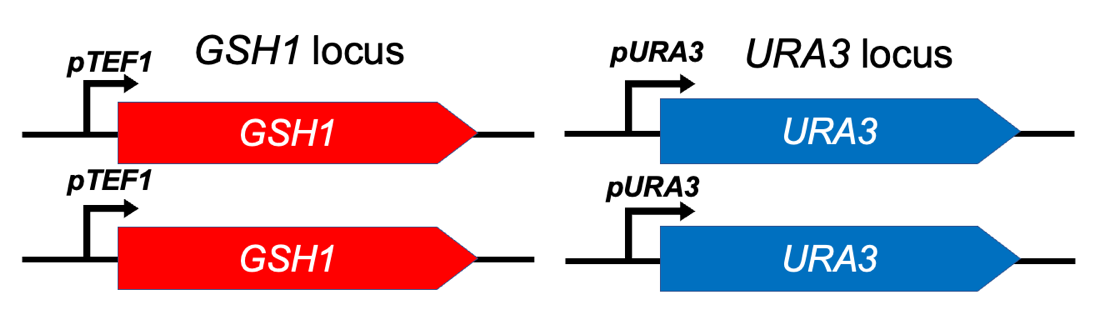


**C.**


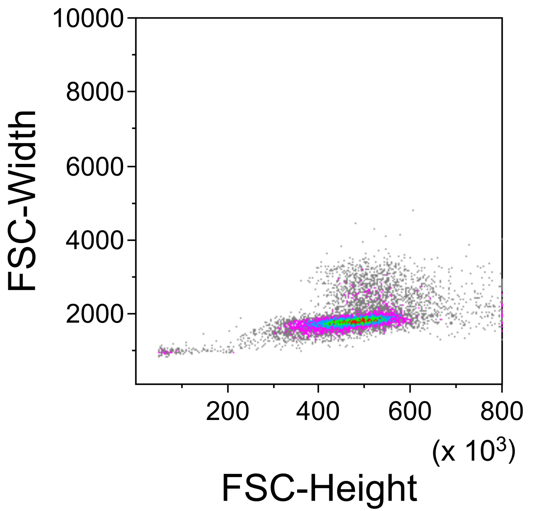

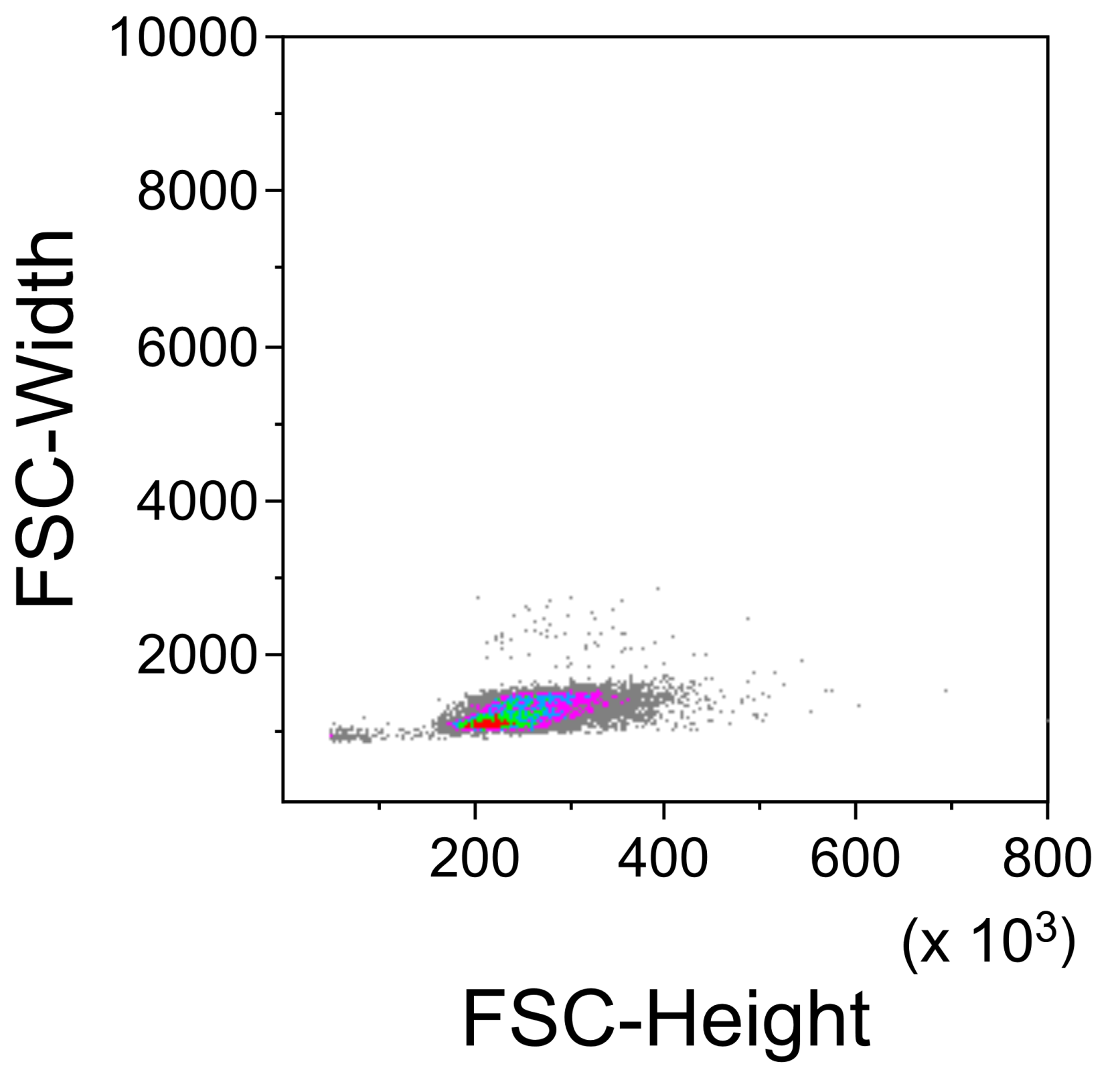

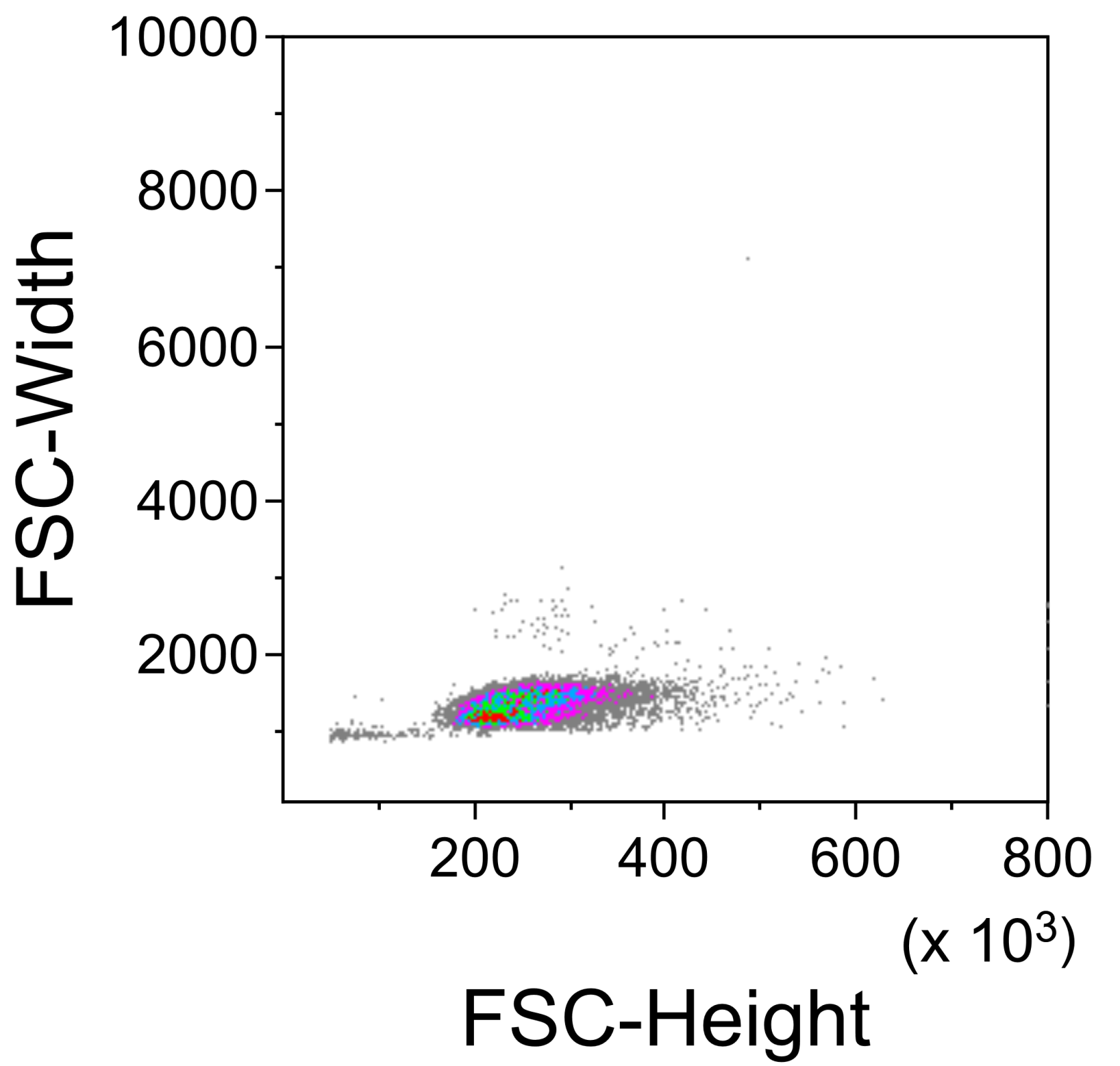


**A.**

**B.**

**C.**

150 µg/ml 5-FOA

100 µg/ml 5-FOA

Control

**Figure S2: Effect of 5-FOA on cellular GSH content and growth of the engineered *S. cerevisiae* containing the heterozygous *GSH1* locus with *URA3 as* counter-selectable marker.** (A) GSH contents of the engineered strain (with the heterozygous *pGSH1* locus shown in Fig. S1A) after cultivation for 24 hours in YNB medium supplemented with 5-FOA at the indicated concentrations. Data represent mean values ± SEM of four biological replicates. p < 0.001 (***), p < 0.0001 (****); ns: not significant. (B) Singlet discrimination by flow cytometry; single cells are in the rectangular gate while cell clumps are outside of the gate according to FSC-height vs FSC-width. ‘Control’, cells cultured without 5-FOA. (C) Growth curves at different concentrations of 5-FOA. ‘Modified strain’ as described in (A) above.

**Figure S3: Long term cellular GSH level and genotype under 5-FOA selection, for modified *S. cerevisiae* containing the heterozygous *GSH1* locus with *URA3* as counter-selectable marker.** (A) GSH content of the engineered strain (with the heterozygous *GSH1* locus shown in Fig. S1A) during sequential batch culture at 100 µg/ml 5-FOA. Control, cells cultured without 5FOA. Data represent mean values ± SEM of three biological replicates. Statistical significance: p < 0.01 (**), p < 0.001 (***), p < 0.0001 (****); ns: not significant. (B) PCR checking of the *GSH1* locus in 10 test colonies (lanes 1 to 10) derived from cultures grown for 50 generations with 100 µg/ml 5-FOA. Leftmost lane, 1 kb DNA ladder. Genotypes unchanged from the starting strain would give two bands, of ~2.5 kb (denoting the *gsh1*Δ::*URA3-GFP* allele) and ~4.5 kb (the *pgsh1*Δ::*HIS3-pTEF1* allele).


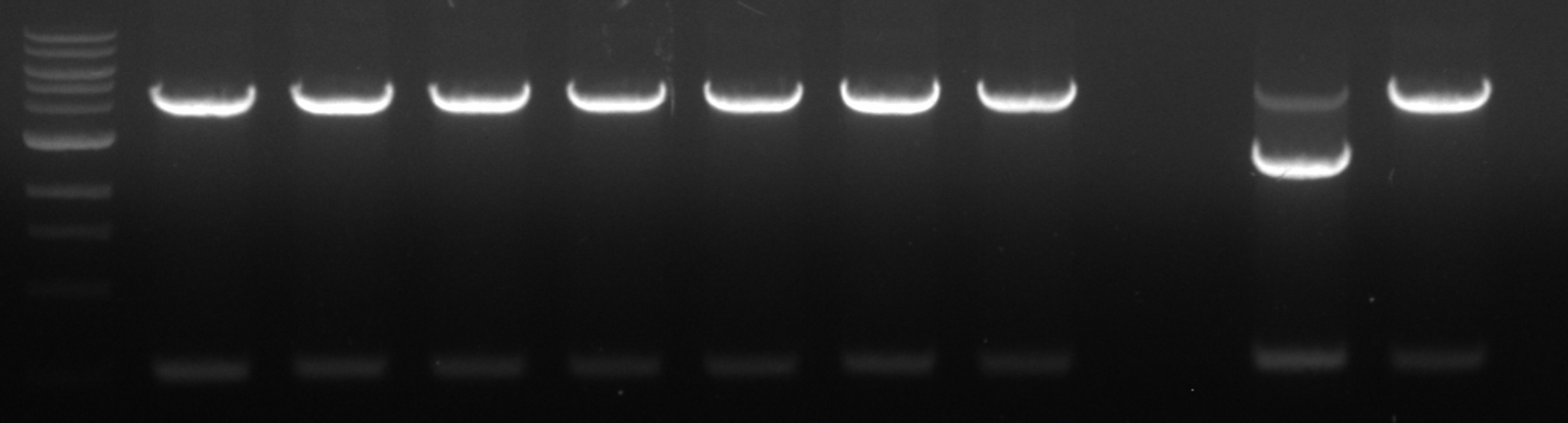


4 kb -

3 kb -

2 kb -

1

2

3

4

5

6

7

8

9

10

**A.**

**B.**

**A.**

**B.**

**C.**

**Figure S4: Cellular GSH level and *URA3* expression under 5-FOA selection for *S. cerevisiae* containing homozygous constructs at the *GSH1* and *URA3* loci.** The relevant modified strains with no 5-FOA addition were used as the controls. (A) Long term cellular GSH level of the homozygous *pGSH1-URA3* strain (as shown in Fig. S1B), grown with daily sub-culture after every ~10 generations to fresh medium ± 100 µg/ml 5-FOA*.* The bar chart represents the mean cellular GSH ± SEM of four biological replicates. (B) Cellular GSH levels of the control *pURA3-URA3* strain (Fig. S1C) and the *pGSH1-URA3* strain. The bar chart represents the mean ± SEM of three biological replicates. (C) Ura3-GFP expression in the homozygous *pGSH1-URA3* strain. The modified strain without additions was used as ‘Control’ while cultivation with 2.5 mM GSH was used as a positive control (addition of GSH should give a reduction of Ura3-GFP expression). GFP fluorescence of cells was measured by flow cytometry. The bar chart represents the mean of three biological replicates, each using the median data of the relevant cell sample. Data were normalized by subtracting the background signal determined for wild type *S. cerevisiae* BY4743. Statistical significance: p < 0.05 (*), p < 0.01 (**), p < 0.001 (***), p < 0.0001 (****).
